# Advocating the potential of artificial intelligence for syndrome discovery in syndromic surveillance systems: A scoping review

**DOI:** 10.1016/j.isci.2026.115103

**Published:** 2026-02-20

**Authors:** Ana Paula Gomes Ferreira, Aleksandar Anžel, Alexander Ullrich, Georges Hattab

**Affiliations:** 1 Robert Koch Institute, Center for Artificial Intelligence in Public Health Research (ZKI-PH), 13353 Berlin, Germany; 2 Freie Universität Berlin, Department of Mathematics and Computer Science, 14195 Berlin, Germany; 3 Robert Koch Institute, Infectious Disease Epidemiology Department, 13353 Berlin, Germany

**Keywords:** Health sciences, Medicine, Health informatics

## Abstract

Syndromic surveillance systems monitor and detect diseases in real time using existing public health data, with syndromes—sets of clinical characteristics—being central to these systems. However, defining these syndromes is a complex and time-consuming task that requires a high level of expertise and the analysis of large, often multimodal and high-dimensional datasets. This challenge is exacerbated by the emergence of new diseases, e.g., due to climate change, urbanization, or globalization. Artificial intelligence has the potential to revolutionize the process of syndrome definition in syndromic surveillance. A scoping review following the PRISMA extension guidelines was conducted to examine the role of artificial intelligence in this field. The PubMed, Google Scholar, OpenAlex, Semantic Scholar, ConnectedPapers, Web of Science, and Embase databases were reviewed. The initial search yielded 2,228 references. After removing duplicates and references outside the scope of the review, 15 studies were included. Most current solutions focus on known syndromes through supervised machine learning, primarily using English free-text data. Artificial intelligence offers immense potential, particularly through open science practices in machine learning, which can accelerate adoption and improve outcomes. However, significant challenges remain to fully realize these benefits. The review concludes with eight strategic recommendations for advancing AI-assisted syndrome discovery in public health. These recommendations address methodology, data handling, collaboration, and standardization to promote effectiveness and global applicability.

## Graphical abstract

**Figure:**
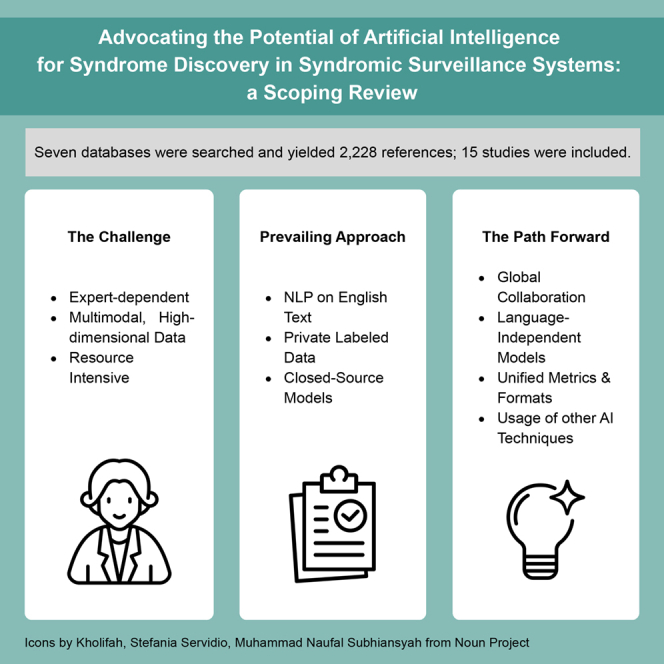

## Introduction

The early detection of disease, often before formal diagnosis, in real or near-real time, is the primary goal of a Syndromic Surveillance System (S3s).^1^^,^^2^

Such a system can collect data from multiple sources, such as social media,^3^^,^^4^ search queries,^5^ and primary health care records.^6^ Syndrome definitions play a critical role in identifying diseases by representing a set of symptoms and characteristics that may indicate a particular condition. The creation of new syndromic case definitions can fall into one of the following scenarios: 1) generating syndromic definitions for existing diseases that do not yet have a specialized definition, or 2) identifying and characterizing previously unrecognized patterns of symptoms or health conditions that do not fit into existing disease classifications, potentially indicating a new syndrome. The process requires careful analysis of available health data and specialized clinical knowledge in both cases.

Syndrome definitions derive from case definitions,^7^ a high-level description of a public health threat. The creation process of a syndrome definition may require defining the scope, refining, and getting approvals.^8^ Developing these definitions can be time-consuming and error-prone, especially in resource-limited settings. The lack of consensus in case definitions can arise between countries, states, and international and national organizations, compelling each organization to work on its own definition.^9^^,^^10^ This hinders collaboration and limits the development of broadly applicable AI-based automation solutions, despite similarities in data and techniques across systems. In addition, the volume of data in healthcare is increasing at an exponential rate,^11^ creating challenges not only for data handling but also for data visualization.^12^^,^^13^

Today, artificial intelligence (AI) plays an essential role in analyzing large amounts of data to identify patterns. The field has been on the rise for the past couple of decades, transforming every industry, with computing power limitations of the past no longer being an obstacle.^14^ AI is increasingly being used in public health,^15^ including genome sequencing,^16^ autism spectrum disorder identification,^17^ face mask-wearing discourse identification,^18^ and mechanical ventilation prediction.^19^

The field of AI has evolved to encompass traditional machine learning (ML) and deep learning (DL). As part of both, natural language processing (NLP) is one of its key applications. Traditional ML consists of techniques that autonomously learn by improving from previously viewed data without explicit programming.^20^

Deep learning, defined by multi-layer artificial neural networks,^21^ is a subset of ML characterized by its architecture and the ability to work with other data representations unsuitable for most traditional ML models.

ML includes four different approaches, each suitable for several scenarios and data: supervised, unsupervised, semi-supervised, and reinforcement learning.^22^ While supervised approaches rely on the data being labeled with the desired output (e.g., confirmed cases of a particular disease), unsupervised approaches do not require labels to be present. A mixture of both approaches constitutes a semi-supervised approach, where the data are partially labeled. When feedback is provided to the ML algorithm during the learning phase, it is considered a reinforcement learning method. Just as medical researchers distinguish novel symptom clusters to define new syndromes, AI researchers employ techniques such as contrastive learning^23^ and causal representation learning^24^ to recognize and interpret unfamiliar data patterns in unseen data. This parallels the importance of robust pattern recognition and categorization in medical diagnostics and AI. It also highlights the potential for cross-disciplinary insights to advance both fields.

Many approaches have been developed to discover new syndromes by analyzing keywords found in syndrome definitions.^25^^,^^26^^,^^27^ However, keeping the definitions up-to-date and defining rare or unknown diseases remains a challenge.^28^^,^^29^^,^^30^

Given the complexity of syndromic surveillance, AI lends itself to various tasks, particularly identifying syndromes from existing data sources. To explore this further, our scoping review aims to answer the questions: *How is AI being applied to the discovery of syndromes in S3s, and what are the gaps and opportunities in this field?*

This article contributes to the field of AI for syndrome discovery by providing a comprehensive review of current AI applications for this task and proposing future directions for leveraging novel AI techniques to improve syndrome discovery and preparedness for emerging health threats. We highlight the need for more flexible and generalized computational solutions, emphasize the importance of open science practices and data sharing, discuss the geographical imbalance in research output, and advocate for a diverse set of learning types and AI techniques for syndrome discovery.

## Methods

Between December 14, 2023, and March 19, 2024, we conducted a scoping review of the literature without date constraints, utilizing Google Scholar,^31^ PubMed,^32^ Semantic Scholar,^33^ OpenAlex,^34^ and ConnectedPapers.^35^ The search was subsequently updated between December 1 and December 30, 2025, to include the Web of Science^36^ and Embase^37^ databases. The selected publications were systematically reviewed, focusing on the usage of AI for syndrome discovery. A protocol outlining the methods and objectives of this scoping review was registered under the same title^38^ on the Open Science Framework (OSF)^39^ to enhance methodological transparency and reproducibility. Although the protocol was registered retrospectively after data synthesis had commenced, the registration details the planned procedures for study identification, screening, data extraction, and categorization, in alignment with scoping review best practices. To ensure the review reflects the most current and comprehensive literature, the search was re-executed prior to submission, as the initial search was conducted one year prior. This update involved running a comprehensive version of the search string and expanding the data sources to include Web of Science and Embase.

We first identified the research questions to guide this review (Table 1). The research questions were organized into the research context, data, and AI techniques.

**Table 1:** Research questions for the scoping review of artificial intelligence techniques in syndrome discovery

Category	Research questions
Research Context	1. Is the research aimed at specific or non-specific syndromes?
	2. From which countries does the proposed research originate?
Data	3. What are the datasets and features used?
	4. Is the data structured or unstructured?
	5. Are the research artifacts open source or open data?
Approach and Technique	6. Which artificial intelligence technique is used?
	7. Which approach is adopted: supervised, unsupervised, semi-supervised, or reinforcement learning?
	8. Which evaluation is used?

This table presents eight key research questions organized into three categories: research context, data, and approach and technique.

Then, we defined the stages and criteria for including or excluding publications. To better refine the desired topics, the selection of publications went through the following four stages.

1. **Search**: We conducted a scoping review by searching multiple databases using query tools and target keyword filtering. (*c.f.*, Algorithm 1).
  Algorithm 1 **Search String Query**
  “syndromic surveillance” AND
   ((syndrom∗ AND (“definition” OR “discovery” OR “classification”)) OR “case definition”) AND
   (“machine learning” OR “artificial intelligence” OR automat∗ OR “deep learning” OR “natural language processing” OR “explainable AI” OR “reinforcement learning”)
2. **Screening**: We removed duplicates and read the publications collected from the previous stage. We discarded the publications that were out of the scope.
3. **Eligibility**: We assessed each full-text, and the research questions were answered.
4. **Inclusion**: We included and noted the AI technique when a research publication used one. The publications that do not make use of an AI technique were discarded.

In the initial stage, the search string algorithm is utilized to choose the articles. The search string comprises three mandatory groups: *syndromic surveillance*, *syndrome discovery*, and *artificial intelligence*. Given that *syndrome discovery* and *disease discovery* are closely related and sometimes used interchangeably,^40^ the search string included *syndromic surveillance* in the first part of it to cover only articles that address this kind of monitoring. The second part of the search string covers *syndrome discovery-related* keywords. It is essential to highlight that *syndrome definition development*, *case definition*, and *syndrome discovery* may represent the same concept: detecting a group of symptoms in an early diagnosis setup.^40^^,^^41^^,^^42^ In the third and last part, multiple terms that refer to AI were used. Even though the prefix *automat* does not belong to the AI field, we notice that many publications use “automated” or “automatic” to represent the usage of AI and keywords (e.g., *machine learning*, *deep learning*, *explainable AI*, and *reinforcement learning* were often omitted from the title and the abstract. The search string is presented in Algorithm 1.

In the fourth and last stage, inclusion criteria were addressed. The scoping review includes English-language research publications that report the development and research of new software solutions for syndrome discovery using AI and clinical data. Publications that used data other than clinical data, such as social media or web queries, were excluded to ensure a better comparison of techniques, data, and features.

The criteria are reported in Table 2.

**Table 2:** Inclusion and exclusion criteria

Included	Excluded
Development of novel techniques for syndrome discovery using artificial intelligence	Statistical techniques, usage of existing software without any modification
Clinical data	Usage of social media, queries, and sensor data
Written in English	Written in languages other than English

The criteria were used in the fourth stage of the scoping review.

## Results

**Figure 1:**
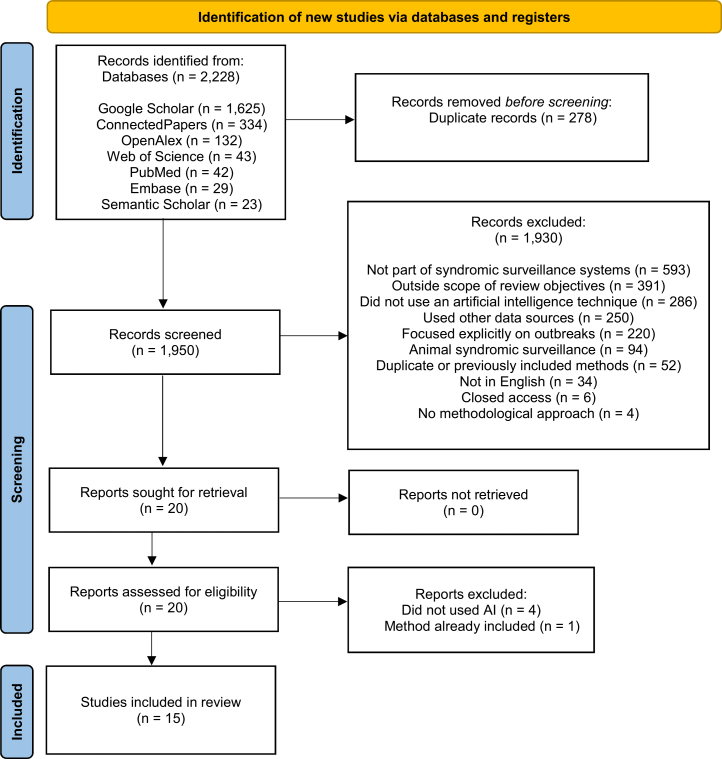
PRISMA 2020 flow diagram shows the selection of studies for the scoping review

In this section, we describe the results from each stage of this review and show our findings for the selected articles. As a result of our search in the databases, 2,228 publications were collected in total, with 1,625 (72.9%) coming from Google Scholar, 132 (5.9%) from Open Alex, 43 (1.9%) from Web of Science, 42 (1.9%) from PubMed, 29 (1.3%) from Embase, 23 (1.0%) from Semantic Scholar, and 334 (15.0%) from Connected Papers. Study selection followed the PRISMA 2020 guidelines. The flow diagram (Figure 1) details the number of records identified, screened, and included. A total of 2,228 records were identified, and 1,950 were screened after duplicates removal. After removing duplicates, 1,930 were screened; 20 were eligible for full-text assessment.

One reviewer thoroughly assessed these publications, and 15 were considered eligible and included in the final list. Table 3 summarizes the reasons for exclusion and the number of articles discarded in each category during the scoping review process.

**Table 3:** Breakdown of excluded articles in the scoping review

Exclusion Category	No. of Publications	(%)	Visualization
Not part of syndromic surveillance systems or irrelevant	593	30.7	–
Outside scope of review objectives	391	20.3	–
Did not use an artificial intelligence technique	286	14.8	–
Used other data sources	250	13.0	–
Focused explicitly on outbreaks	220	11.4	–
Animal syndromic surveillance	94	4.9	–
Duplicate or previously included methods	52	2.7	–
Not in English	34	1.8	–
Closed access	6	0.3	–
No methodological approach	4	0.2	–

This table summarizes the reasons for exclusion and the corresponding number of articles for each category, based on the analysis of 1,930 discarded articles, ordered by frequency. The most prevalent category was elected to represent the article. The visualization column shows a horizontal bar representation of the percentages.

The included publications were evaluated according to the research questions. Each category was analyzed to understand the state-of-the-art practices for discovering new syndromes using artificial intelligence. The following sections present the results obtained from the fully assessed studies related to the research topic.

### The overall research context

From the 15 selected publications shown in Table 4, the earliest was from 2002 and the latest from 2024. From this list, only two (13.33%) techniques aim to discover syndromes that were not seen before (non-specific syndromes). Only three (20%) articles are open-source. The articles included were from the following countries: 8 (53.33%) from the United States of America, 2 (13.33%) from Germany, 2 (13.33%) from Australia, 1 (6.67%) from France, 1 (6.67%) from Italy, and 1 (6.67%) from Pakistan. It is worth noting that the United States is the leading country by far in this ranking, which is consistent with the pattern observed by Musa.^57^

**Table 4:** List of publications focusing on syndrome discovery using artificial intelligence and clinical data

Year	Publication	Specificity	Open Source	Country
2002	Ivanov^43^	Specific	No	USA
2003	Olszewski^44^	Specific	No	USA
2005	Muscatello^45^	Specific	No	Australia
2005	Chapman^46^	Non-Specific	No	USA
2011	Tsui et al.^47^	Specific	No	USA
2012	Gagliardi^42^	Specific	No	Italy
2013	Gerbier-Colomban^48^	Specific	No	France
2015	Muhammad^49^	Specific	No	Pakistan
2018	Tsui^50^	Specific	No	USA
2019	Lee^51^	Specific	No	USA
2021	Wen et al.^52^	Specific	Yes	USA
2022	Nobles^53^	Non-Specific	Yes	USA
2022	Rapp^54^	Specific	Yes	Germany
2022	Wagner^55^	Specific	No	Germany
2024	Khademi^56^	Specific	No	Australia

For each publication, the table describes whether it addresses specific or non-specific syndromes, whether the data and source code are open-sourced, and the year and the country where the work was conducted. The open-source code provided with each publication can be found at the following links: https://github.com/OHNLP/MedTagger,^52^https://github.com/OHNLP/AEGIS,^52^https://github.com/danielbneill/pre-syndromic-surveillance,^53^ and https://github.com/mrapp-ke/SyndromeLearner.^54^

The included publications mainly aimed to differentiate syndromes to prevent new outbreaks, followed by the need for better case detection accuracy^50^^,^^55^ and faster response.^43^^,^^44^^,^^51^^,^^56^ The primary motivators are fighting bio-terrorism^43^^,^^45^^,^^46^ and improving data quality^55^ (e.g., dealing with missing data and showing value in inputting better data into the systems). The limitations of the analyzed articles include the lack of generalization (support for different syndromes or settings) and struggles with the balance between sensitivity and specificity. Prior studies have noted the limited available data^43^^,^^44^ and the lack of a gold standard benchmark.^46^^,^^51^

**Figure 2:**
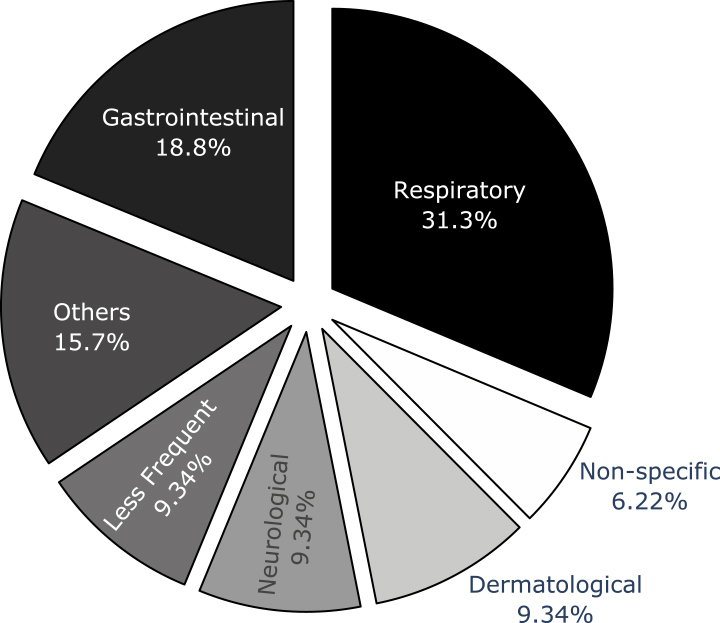
Distribution of 15 publications by targeted syndrome group The chart represents the percentage of publications focusing on different syndrome categories. Respiratory syndromes were the most targeted (31.3%), followed by gastrointestinal (18.8%). The “Others” category (15.7%) includes syndromes that are not representative enough to have their own group or are too generic to belong to only one group (e.g., rash and cough). “Non-specific” (6.22%) represents publications aiming to detect any syndrome, whether predefined or not. “Less Frequent” (9.34%) combines less frequent categories: cardiovascular, injury, and substance-related syndromes. This distribution highlights the focus on respiratory and gastrointestinal syndromes in the studied publications, while also showing a diverse range of other targeted syndromes.

This scoping review also tracked whether an approach targeted known and specific syndromes or unknown and non-specific syndromes. Specific syndromes, such as influenza and dengue, have a definition, while non-specific syndromes can represent any group of symptoms that suddenly appear together.^58^ Of the 15 reviewed publications, 13 focused on known and specific syndromes, with respiratory conditions being the most common, followed by gastrointestinal syndromes (Figure 2). Only two publications^46^^,^^53^ propose techniques for autonomous syndrome identification. S3s often target infectious diseases, but in this work, 50% of the publications targeted non-infectious diseases.

#### Data

The quality of an outcome in an artificial intelligence application will be as good as its data.^59^ For that reason, we aimed to better understand the data sources, features, and nature of them, and whether they are accessible or not. The data sources of the selected publications were emergency department (ED) visits and primary care clinical records, as well as clinical and report evaluations.

The features mentioned by at least two articles are displayed in Table 5, along with the techniques and their references. Chief Complaints is the most used feature when identifying new syndromes and symptoms. Moreover, the International Statistical Classification of Diseases and Related Health Problems (ICD codes) is present in both discharge and triage diagnoses, providing a great universal source of structured medical knowledge information. Out of 15 publications, eleven used structured data to develop their models.

**Table 5:** List of features in the final set of publications shown with the number of appearances in parentheses

Features	No. Publications	Techniques	Reference
Free-text chief complaint	9	Naive Bayes (2), Bigram Bayes, NLP engine, Bayesian network (2), MNB, SVM, LSTM (2), GRU, Neural network, Logistic regression, LDA	Ivanov et al.; Chapman; Gerbier-Colomban; Ali; Tsui; Lee; Wen; Nobles; Khademi^43^^,^^46^^,^^48^^,^^49^^,^^50^^,^^51^^,^^52^^,^^53^^,^^56^
Discharge diagnosis (ICD)	7	Bayesian network, Bayesian classifier, MNB, SVM, LSTM, GRU (2), Neural network (2), Logistic regression (2), LDA, RoBERTa, XGBoost	Olszewski; Gerbier-Colomban; Ali; Tsui; Lee; Nobles; Wagner^44^^,^^48^^,^^49^^,^^50^^,^^51^^,^^53^^,^^55^
Provisional diagnosis (ICD)	7	Naive Bayes (2), Bigram Bayes (2), NLP engine, MNB, SVM, LSTM, and GRU, Logistic regression (2), Rule-learning, RoBERTa, XGBoost	Ivanov et al.; Olszewski; Muscatello; Lee; Wen; Rapp; Wagner^43^^,^^44^^,^^45^^,^^51^^,^^52^^,^^54^^,^^55^
Age	7	Cluster analysis, Naive Bayes, Bayesian network, Logistic regression (2), LDA, Rule-learning, Neural Network, RoBERTa, XGBoost	Gagliardi; Muscatello; Tsui et al.; Gerbier-Colomban; Tsui; Nobles; Rapp^42^^,^^45^^,^^47^^,^^48^^,^^50^^,^^53^^,^^54^
Sex	5	Naive Bayes, Logistic regression, LDA, Rule-learning, Neural Network, RoBERTa, XGBoost	Muscatello; Tsui et al.; Gerbier-Colomban; Nobles; Rapp^45^^,^^47^^,^^48^^,^^53^^,^^54^
Date and time of admission	4	Bayesian network, Neural network (2), Logistic regression (2), LDA, Neural Network, RoBERTa, XGBoost	Gerbier-Colomban; Ali; Tsui; Nobles;^48^^,^^49^^,^^50^^,^^53^
Diseases	3	Cluster analysis, NLP engine, Bayesian network	Gagliardi; Tsui; Wen;^42^^,^^50^^,^^52^
Type of admission	4	Naive Bayes, Logistic regression	Muscatello; Gerbier-Colomban; Rapp; Wagner^45^^,^^48^^,^^54^^,^^55^
Vital parameters (e.g., temperature, blood pressure, and pulse frequency)	2	Rule-learning, XGBoost, Logistic regression, BiGRU, Neural network	Rapp; Wagner^54^^,^^55^
No isolation	1	Rule-learning	Rapp^54^
Disposition	1	Rule-learning	Rapp^54^
Department e.g., Surgery	1	XGBoost, Logistic regression, BiGRU, Neural network	Wagner^55^
Severity	1	XGBoost, Logistic regression, BiGRU, Neural network	Wagner^55^

NLP, natural language processing; MNB, multinomial Naive Bayes; SVM, Support Vector Machine; LSTM, long short-term memory; GRU, gated recurrent unit; LDA, linear discriminant analysis; ICD, International Classification of Diseases.

We assessed whether the solutions presented contained publicly available code (open-source) and/or data (open-data). Only three eligible publications^52^^,^^53^^,^^54^ have their solutions open-sourced, and none are open-data.

#### Artificial intelligence techniques

**Figure 3:**
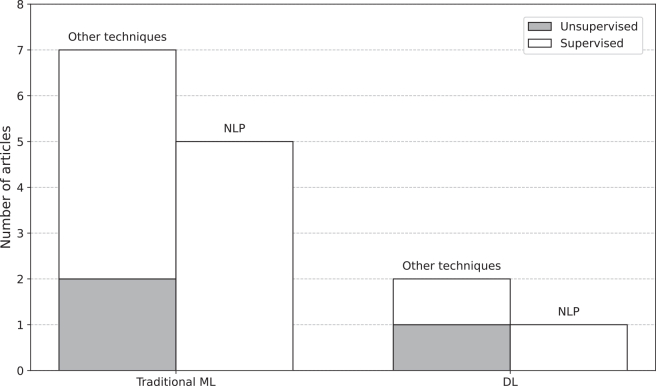
Distribution of publications by machine learning approach and technique The chart compares traditional machine learning (ML) and deep learning (DL) approaches, further categorized into natural language processing (NLP) and other techniques. Each category is subdivided into supervised and unsupervised learning methods. The y axis represents the number of publications.

**Figure 4:**
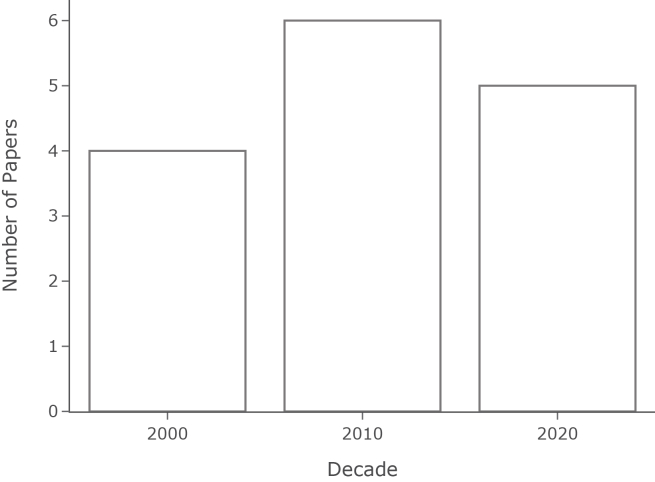
Number of AI-driven syndromic surveillance studies by decade The Figure shows that 4 studies were published in the 2000s, 6 in the 2010s, and 5 in the 2020s.

**Figure 5:**
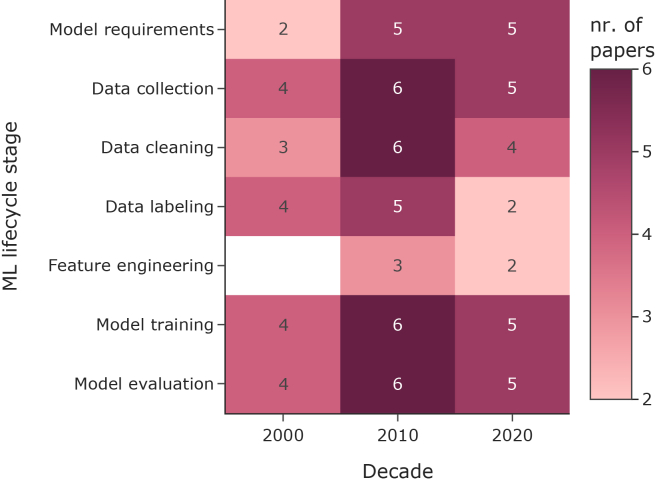
Coverage of ML life cycle stages across studies by decade The heatmap displays the count of articles addressing each stage. The white cells indicate the absence of studies in the given decade for the given stage.

Up to this point, the preceding sections have laid the groundwork for the main focus of this review: presenting an overview of how AI is utilized for syndrome discovery. An overview of the learning methods used to discover new syndromes is crucial to understanding the current state of AI in this field. Our review uncovered only supervised and unsupervised ML techniques, as shown in Figure 3. Out of 15 publications, twelve used supervised and three unsupervised learning, having none that used semi-supervised or reinforcement learning. Figures 4 and 5 present a historical perspective by showing how the articles were distributed over the decades and their lifecycles.

Table 6 offers an overview of the AI techniques presented by each publication compared to the seven-step life cycle of a machine learning model. The seven-step life cycle includes: model requirements, data collection, data cleaning, data labeling, feature engineering, model training, and model evaluation. Additional steps should be considered for validation depending on where the model is deployed.^60^ While this entire life cycle includes model deployment and monitoring, the solutions found in this scoping review were often only prototypes. This seven-step life cycle is adopted to review the related work and to explicitly identify missing steps.

**Table 6:** Selected publications compared by artificial intelligence development workflow

Reference	Model requirements	Data collection	Data cleaning	Data labeling	Feature eng.	Model choice, training and validation	Model evaluation
Ivanov et al.^43^	N/A	16,880 triage diagnoses	N/A	Physician classification	N/A	Naive Bayes, Bigram Bayes; test set validation	Expert evaluation
Olszewski^44^	N/A	28,990 Utah ED diagnoses	Lowercase, punctuation removal	Physician classification	N/A	Unigram, bigram, word pairs; 10-fold cross-validation	ROC
Muscatello^45^	Automated, broad-based, real-time	12 EDs, 1.2M visits/year	Text processing, spell-checking	ICD-10 code assignment	N/A	Bayesian classifiers	Correlation and sensitivity
Chapman^46^	Use ubiquitous free-text complaints	Pittsburgh hospital ED	Abbreviation mapping, spell-checking	Manual syndromic labeling	N/A	Bayesian network	ROC, AUC
Tsui^47^	Address existing limitations	UPMC ED reports	De-identification	PCR tests, keywords	NLP symptom extraction	Bayesian network, Expectation Maximization-Maximum-A-Posteriori	ROC, AUROC
Gagliardi^42^	N/A	UCI Dermatology dataset	Removed missing values	N/A	N/A	Cluster analysis optimized by a genetic algorithm using a scoring function	Qualitative, Disease Addressing Index
Gerbier-Colomban^48^	Early infectious detection	10,895 ED patients, France	Text processing, categorization	Discharge diagnoses as the gold standard	N/A	Logistic regression; cross-validation	ROC
Ali^49^	Disease patterns and seasonality specific to Pakistan	2011-2013 Lahore hospital data	Error and missing value processing	ICD-10 code assignment	N/A	Neural network, 40 neurons; 40-15-45 split validation	Cross-entropy error, sensitivity, specificity, ROC, classification accuracy
Tsui^50^	Public health, clinical support	ED HL-7 records	Duplicate removal, number of inflammation signs, and age categorization	Manual influenza labeling	31 clinical findings	Bayesian network	Test dataset comparison
Olszewski^51^	Real-time monitoring	3.6M ED visits, NYC	Text cleaning, ICD mapping	Manual syndromic labeling	Unigrams, bigrams, word embeddings	MNB, SVM, LSTM, GRU; 60-20-20 split validation	F1 scores
Wen^52^	Unsupervised learning, historical validation	Mayo Clinic, 2011-2020	Weekends, holidays, and invalid symptoms data removal	N/A	N/A	NLP engine; 70-30 split, cross-validation	Comparison to actual flu data
Nobles^53^	Overcome existing limitations	28M ED cases, NYC, 2010-2016	Medical language processing, spell-checking	N/A	N/A	Emerging Topic LDA Model	Blinded studies with practitioners
Rapp^54^	A data-driven approach to discover disease patterns in historic data	1.9M German ED cases, 2017-2021	N/A	N/A	N/A	Rule-learning algorithm	Synthetic and real-world syndrome definitions
Wagner^55^	Impute missing diagnosis codes; ILI syndrome prediction	384,021 ED visits	Vital aggregation, ICD truncation	Rule-based ILI definition	Temporal features, hierarchical inputs, BoW, LSTM embeddings	NB, DataWig, LogReg; 80-20 split, SMOTE resampling	F1, precision, recall, specificity, Pearson’s r, qualitative review
Khademi^56^	Real-time febrile convulsion detection	76k triage texts	Anonymization, contraction, and expansion	Manual annotation, synthetic augmentation	SQL pattern matching, TF-IDF, Word2Vec, RoBERTa embeddings	XGBoost, LogReg, BiGRU, CNN, RoBERTa	Precision, Recall, F1, real-world audit

ED, Emergency Department; ICD, International Classification of Diseases; ROC, receiver operating characteristic; AUC, area under the curve; UPMC, University of Pittsburgh Medical Center; PCR, polymerase chain reaction; NLP, natural language processing; AUROC, area under the receiver operating characteristic; UCI, Irvine, University of California; HL-7, Health Level Seven; NYC, New York City; MNB, Multinomial Naive Bayes; SVM, Support Vector Machine; LSTM, long short-term memory; GRU, gated recurrent unit; LDA, latent Dirichlet allocation.

None of the models reported by the selected studies had an explicit model requirements phase. It was clear that the primary goal of discovering syndromes for better reporting in their S3s was shared. However, from the context provided in the articles, we could see that some share the need to detect the performance of existing systems in terms of precision and efficiency. Another need was to overcome the difficulty of relying on often missing ICD diagnostic codes and use free-text triage chief complaints as the main feature. These requirements emphasize the need for an automated, efficient, and broad-based system that can provide early detection of infectious diseases using readily available data. There are no details on how the data are collected. The data utilized was private, except in the case of the dataset presented by Gagliardi.^42^

In the data cleaning stage, there were tasks involving text preprocessing (e.g., removing duplicates and lowercasing) and mapping to structured domain-specific data such as the International Statistical Classification of Diseases and Related Health Problems (ICD). As the name states, the data labeling phase comprises labeling the data with the desired syndromes. Experts were involved in half of the publications that reported data labeling. Only five publications explored the feature engineering process, three of which were for symptom extraction.

The selected studies use a variety of models, including neural networks, Bayesian networks, logistic regression, and more advanced techniques such as long short-term memory (LSTM) and gated recurrent units (GRUs). More than half of the studies (60%) utilize Bayesian approaches (Naive Bayes, Bigram Bayes, Bayesian classifiers, Bayesian network, Multinomial Naive Bayes). These approaches handle text well with text and address some of the challenges reported, such as handling missing data and others inherent to symptoms data, which is often complex and hierarchical. To validate their models during the development process, techniques such as train-validation-test splits^43^^,^^51^^,^^52^ and cross-validation^44^ were used. Rapp^54^ uses rule-learning to identify disease patterns in the data, being the only one from this category in the selected list.

It is essential to examine how the techniques used in S3s perform to avoid false positives. The publications that modeled the surveillance problem as a regression one mainly utilized correlations and comparisons to actual data (e.g., flu data in work by Wen et al.^52^). Others that modeled it as a classification problem utilized a variety of evaluation methods, including Receiver Operating Characteristic (ROC) curves and Area Under the ROC Curve (AUC/AUROC),^44^^,^^46^^,^^47^ sensitivity and specificity measures,^45^^,^^49^ F1 scores,^51^^,^^56^ and classification accuracy.^49^ Rapp^54^ evaluated their algorithm using synthetic syndromes of varying complexity to assess its reconstruction capabilities in a controlled environment. The articles that utilized unsupervised methods reported evaluation techniques such as qualitative evaluation and Disease Addressing Index for cluster analysis,^42^ comparison to actual flu data,^52^ and blinded studies with practitioners.^53^

## Discussion

**Figure 6:**
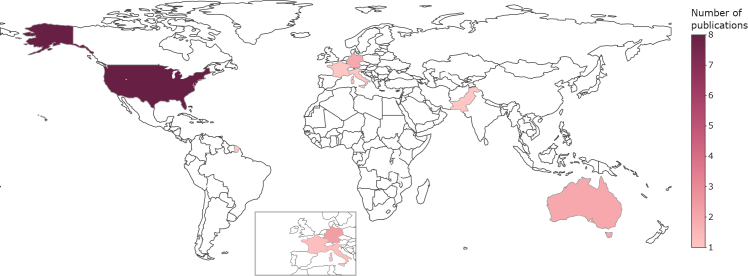
Global distribution of selected publications by country This choropleth map illustrates the geographical distribution of scientific publications in different countries. The color intensity represents the number of publications, ranging from 1 (lightest) to 8 or more (darkest), as indicated by the color palette. The United States has 8 publications, Australia and Germany have 2, France, Italy, and Pakistan each have 1.

First, according to our review, the most common AI approach to syndrome discovery is an NLP engine that classifies English words from chief complaints to respiratory syndromes using labeled but private data and closed-source code. Our review of AI applications in syndromic surveillance systems reveals several critical insights and challenges in the field. The findings focus primarily on supervised learning techniques and specific syndromes, particularly respiratory diseases. While this focus is consistent with prioritizing known infectious diseases that pose significant public health risks, it may inadvertently leave systems underprepared for novel or emerging health threats. The heavy reliance on English-language free-text data, primarily from chief complaints, introduces a strong language dependency that may limit the global applicability of these systems. In addition, we identified notable gaps in the development pipeline, including a need for more flexible and generalized solutions and limited exploration of advanced AI techniques. A significant gap in open science practices was also evident, with a lack of publicly available code, data, and standardized benchmarks hindering collaborative development and reliable evaluation of different solutions. These findings, along with a significant geographic imbalance in research output, underscore the need for more comprehensive, adaptive, and globally collaborative approaches to using AI in syndromic surveillance, as well as a greater commitment to open science principles. A recent publication by Schmallenbach^61^ examined the global geography of AI in life sciences research and found that while Asia leads in total publications, North America and Europe contribute most of the AI research that appears in high-ranking publishers and generates more citations. This is consistent with our findings on the geographic imbalance in AI applications for syndromic surveillance (Figure 6), highlighting the need for more diverse and globally collaborative approaches in this area.

Second, our results show a strong focus on supervised learning techniques and specific syndromes, particularly respiratory diseases. This emphasis is likely due to the prioritization of known infectious diseases that can affect large portions of the population and cause significant morbidity and mortality. The respiratory syndrome group is the top target for syndrome discovery, as shown in Figure 2. However, this approach may leave systems unprepared for novel or emerging health threats. Historical data have proven to be the key to predicting new outbreaks.^62^ But how do we prepare for the unknown? And how do we do it quickly? Building solutions that can capture different symptoms with little or no human intervention in S3s is overlooked. Some syndromic groups carry syndromes with overlapping symptoms, while other groups do not, which can be a challenge when building models without labeled data. The World Health Organization (WHO) has included “Disease X” as a placeholder in its disease priority list for pathogens that are not currently known to cause human disease.^63^ There is great potential in developing more flexible unsupervised learning techniques to discover emerging and known syndromes.

Third, the reliance on free text, especially chief complaints, as the primary feature is notable. This feature is used by 9 of the 15 studies. While this approach provides flexibility to adapt to new conditions relevant to public health, such as heat-related illnesses^64^ or motor vehicle crashes,^65^ it also introduces a strong language dependency, with most solutions centered on English-language data. This language focus may limit the global applicability of these systems. Currently, open-source multilingual models,^66^^,^^67^ including those trained on domain-specific data such as medical texts, present promising opportunities to address language bias and geographical imbalances. These models can help bridge the gap for underrepresented languages and regions, especially in healthcare, where access to annotated data are often limited. Libraries such as Sentence Transformers^68^ facilitate the development of semantic representations across various languages, allowing for more inclusive and globally applicable natural language processing (NLP) applications. However, deploying AI systems for underrepresented languages and regions raises significant ethical considerations. These include the risk of exacerbating existing disparities, the necessity for community involvement in data collection and validation, and the importance of transparent and accountable model development. Ensuring fairness and cultural sensitivity in these contexts is essential for creating equitable AI solutions.

Fourth, the review also reveals gaps in the development pipeline for these AI solutions. While the studies emphasize the need for real-time solutions and accuracy in identifying disease patterns, similar data sources, and characteristics, there is a notable lack of flexible and generalized solutions. The data from the studies in this review were not combined with other sources, providing an opportunity to explore more multimodal learning capabilities. We also found that many approaches used free text to identify symptoms and disease codes, often repeating tasks such as text cleaning and ICD mapping. This is another opportunity to empower practitioners to develop open source solutions to implement repeated tasks on similar data, a need that is becoming increasingly urgent. During the data labeling process, 6 of the 15 studies used human labeling, a step that could be skipped if open datasets were available. Only 5 publications used feature engineering, which could have significantly improved their results and accuracy—making it a missed opportunity to fully leverage the potential of their datasets.

Fifth, an interesting finding is the prevalence of Bayesian approaches in the reviewed studies. These methods appear to be well-suited to handle the complex, hierarchical nature of syndromic data and to address challenges such as missing information. Despite the dominance of supervised learning in AI-driven syndrome discovery, several underrepresented machine learning techniques offer valuable yet largely untapped potential. For instance, transfer learning could greatly enhance model generalization, especially in global health contexts where syndromic patterns—such as respiratory or gastrointestinal clusters—often recur across countries. Pretrained models on large clinical corpora have been shown to improve downstream performance even when labeled data is scarce or domain-shifted.^69^^,^^70^ Likewise, semi-supervised learning could help mitigate the high cost and limited availability of labeled clinical data, leveraging large volumes of unlabeled health records to improve classification performance.^71^ However, these approaches remain underused due to structural challenges such as overlapping symptoms across syndromes, privacy constraints that limit data sharing,^72^ and a shortage of machine learning expertise in many public health settings.^73^ Reinforcement learning poses further complexity, requiring dynamic feedback environments that are rarely feasible in static or retrospective clinical datasets. Meanwhile, emerging techniques such as contrastive learning, which have demonstrated strong performance in biomedical representation learning,^74^ remain virtually unexplored in this domain. A broader adoption of these advanced techniques—coupled with privacy-preserving infrastructure and international collaboration—could significantly accelerate innovation in syndrome discovery. There is a wide range of scoring techniques, from expert classification to more statistical methods such as ROC curve analysis and AUROC. Nevertheless, the unsupervised approaches rely on practitioner evaluation^52^^,^^53^ and custom metrics^42^ to evaluate the results, suggesting a need for the evaluation standardization of unsupervised learning models in this domain. While many studies focus on model development and initial evaluation, there is little discussion of deployment strategies or long-term monitoring of model performance in real-world settings. This gap may reflect the prototypical nature of many solutions, but it also highlights the need for more comprehensive, end-to-end approaches to implementing AI in syndromic surveillance.

Sixth, this scoping review attempts to collect as many related publications in the field of AI for syndrome discovery as possible, but some publications may be missing. An important consideration is the close interconnection between syndrome discovery and outbreak detection in the existing literature. In many studies, the definition or refinement of syndromes has been developed primarily as a component of outbreak detection methods, rather than as an independent research objective. As a result, the number of publications explicitly dedicated to syndrome discovery is limited. This methodological overlap explains why our synthesis identified a relatively small set of eligible studies and highlights the need for clearer distinctions in future work between these closely related but conceptually distinct tasks. Another consideration to make is that the stages outlined are similar to those in a regular software engineering development cycle. However, we acknowledge that some of the identified stages may not have been explicitly disseminated or formalized when specific solutions were proposed. The evolution of the field has been organic, and our table organization attempts to structure that evolution retrospectively.

Seventh, while the review highlights a significant gap in open science practices in the field, the lack of publicly available code, data, and standardized benchmarks hinders collaborative development and reliable evaluation of different solutions. This is an opportunity to create tools that address a global problem as a community, working together. The use of synthetic data needs to be explored, and federated learning could be an alternative to share knowledge and experience on how models behave in different data settings. Creating gold standard datasets can be the starting point for developing new solutions in an area where data are scarce. Using openly available datasets can provide a practical and impactful starting point for developing and evaluating models.^75^^,^^76^ Brazil’s OpenDATASUS, one of the largest publicly available clinical data repositories worldwide,^75^ offers extensive health data that enables large-scale epidemiological and clinical research. Similarly, in smaller scale, MIMIC-IV^76^ provides rich, de-identified hospital data widely used in benchmarking and model development. These datasets allow researchers to explore model performance across diverse healthcare settings.

Eighth and finally, the review points to a significant geographical imbalance in research output, with most studies (61.54%) coming from the United States. This concentration may limit the diversity of approaches and potentially result in less adaptable solutions for different healthcare systems and populations.

Ultimately, we believe that AI has the potential to revolutionize healthcare; however, high implementation costs and the scarcity of a specialized workforce present significant hurdles, as further elaborated in our conclusion. To bridge the gap between current experimental models and real-world utility, syndrome discovery must leverage AI/ML not only for detection but also to support decision-making regarding surveillance indicators and the evaluation of public policy performance. This evolution requires the adoption of more modern technology stacks and a synergistic cooperation between humans and AI. The fifteen studies reviewed here offer an initial glimpse into AI-driven syndromic surveillance. However, larger and more diverse bodies of research supported by these modernized frameworks are needed before definitive best practices can be established.

### Limitations

Our scoping review methodology includes several important caveats that should be taken into consideration:

We may have missed relevant publications despite using multiple bibliographic databases and a broad search string. Terminology in this field is highly heterogeneous—terms such as “syndrome discovery,” “disease discovery,” and “case definition” are used inconsistently, which could lead to retrieval bias.

We restricted our inclusion to English-language research, which may underrepresent research from under-resourced regions.

A scarcity of publicly available code, datasets, and gold-standard evaluation benchmarks in the underlying literature hampered our ability to compare methods uniformly. As a result, our synthesis may be biased toward better-documented approaches with openly shared artifacts.

We did not account for temporal heterogeneity in the included studies. Over the past two decades, advances in coding standards (e.g., ICD versions), clinical workflows, and computational resources mean that early work may not be directly comparable to more recent approaches. This lack of historical contextualization could influence interpretations of longitudinal trends.

### Recommendations

In our discussion section, we explored different gaps and opportunities brought by AI for the discovery of syndromes. Later in discussion, we list our recommendations for the future of the field. While implementing these recommendations may face challenges, such as achieving consensus on case definitions and maintaining data quality, it is important to remember that we are part of a collective effort. This effort aims to advance syndromic surveillance and improve rapid response to emerging health threats, making these recommendations worthwhile endeavors. Realizing the full potential of AI-assisted syndrome discovery in public health necessitates a paradigm shift toward collaborative, interdisciplinary, and globally inclusive research practices.^15^ By embracing these recommendations, we, as a scientific community, can work toward more robust, adaptable, and effective S3s, ultimately enhancing our global capacity to detect and respond to public health threats.

1. **Promote Open Science practices (collaboration)**: To foster collaboration and accelerate progress, we advocate for creating and maintaining public repositories of anonymized health data, developing synthetic datasets for algorithm testing and benchmarking, establishing a culture of open-source development in the field, implementing federated learning techniques to share knowledge while preserving data privacy, and publishing models while being open to collaboration. By embracing open science practices, we can promote broader technology sharing and reduce inequities in access.^77^ This approach helps disseminate advanced development pipelines, ultimately accelerating innovation.^78^ These practices directly address several challenges identified in this review, specifically the fourth challenge (gaps in the development pipeline), the fifth challenge (limited exploration of more advanced AI techniques), the seventh challenge (lack of open science practices), and the eighth challenge (geographical imbalance in research output). We hope that increasing the adoption of open science practices will encourage further work in this field, as noted in our sixth observation.
2. **Establish a standardized syndrome format (standardization)**: We propose creating a centralized, open-access knowledge base of syndrome definitions and characteristics in a standardized format. This knowledge base would be valuable for researchers, healthcare professionals, and AI developers working in syndromic surveillance. It would facilitate improved AI model development, enhance transfer learning capabilities, and promote global collaboration in public health by providing consistent, well-structured data on various syndromes, including their symptoms, temporal patterns, and relationships. A standardized format facilitates interoperability^79^^,^^80^ between systems and enables fair comparisons between health systems and countries. This recommendation addresses the seventh challenge (gap in open science practice) and the eighth challenge (geographical imbalance in research output) identified in this review.
3. **Foster collaboration between Global North and South public health organizations (collaboration)**: To ensure the global applicability of AI solutions in syndromic surveillance, we suggest fostering international collaborations^81^ and research partnerships,^82^ providing funding and resources for syndromic surveillance research in underrepresented regions, and developing adaptable solutions that can be easily customized for different healthcare systems. This recommendation addresses the eighth challenge (geographical imbalance in research output) identified in this review.
4. **Address language dependencies (linguistic/data-related)**: To overcome the current English-language bias, we suggest creating multilingual datasets and models,^67^ developing language-agnostic features for syndrome classification, and collaborating with international partners to diversify data sources. This recommendation directly addresses the eighth challenge (geographical imbalance in research output) identified in this review.
5. **Expand AI technique exploration (methodology)**: We propose investigating deep learning^83^^,^^84^ and transfer learning applications^85^ in syndromic surveillance and organizing challenges or hackathons to encourage innovation in AI for public health. Transfer learning is particularly promising for syndrome discovery because it allows us to leverage existing knowledge about known health conditions to identify new or emerging threats more quickly and efficiently. This recommendation addresses the first (focus on NLP, English language, and supervised learning), second (historical usage of supervised learning and specific syndromes), third (free-text reliance), and fifth (limited exploration of more advanced AI techniques) identified in this review.
6. **Standardize evaluation metrics (methodology)**: To enable reliable comparison of different approaches, we recommend developing or using standardized metrics^86^^,^^87^ for evaluating unsupervised syndrome discovery and creating benchmark datasets for comparing different methods. Specific metrics, tailored to evaluate syndrome discovery, enable a fair comparison among different solutions. This recommendation touches the fourth (focus on NLP, English language, and supervised learning) and fifth (limited exploration of more advanced AI techniques) challenge identified in this review’s observations.
7. **Diversify learning approaches (methodology)**: Our review shows the overuse of supervised learning and natural language processing techniques, which are not replicable in most realities. An alternative to this is exploring other learning solutions such as unsupervised learning techniques^88^^,^^89^ or semi-supervised learning,^90^ where the data can be partially labeled (perhaps using diagnosed data or lab results). This recommendation connects to the first (focus on NLP, English language, and supervised learning), second (focus on NLP, English language, and supervised learning), and fifth (limited exploration of more advanced AI techniques) challenge identified in this review’s observations.
8. **Promote shared tools (technical/process-oriented)**: To streamline the development process and improve model performance, we recommend establishing open-source libraries for common tasks^91^ (e.g., text cleaning and ICD mapping), exploring multimodal learning by integrating diverse data sources and investing in feature engineering techniques that incorporate data from various domains, such as weather patterns and expert publications. This recommendation is related to the fourth (focus on NLP, English language, and supervised learning), fifth (limited exploration of more advanced AI techniques), seventh (gap in open science practice) observations, and eighth challenge (geographical imbalance in research output).

### Conclusion

S3s aim to promptly inform about potential public health threats, making artificial intelligence the perfect approach for precise real-time detection. This scoping review aimed to shed light on using AI for syndrome discovery (SD) using clinical data in S3s as a key component. Our results show a significant overlap in data sources and features, with a large focus on the same group of syndromes. This is an indicator that SD solutions can be built collaboratively. However, the lack of golden standards, open data, and open source solutions in this area hinders rapid development and reliable evaluation of different solutions. Privacy concerns are often raised when combining clinical data and open data. Still, they can be addressed using synthetic data for dataset creation and training federated learning models. Leveraging novel AI techniques in syndrome discovery remains unexplored, but it holds great potential to improve precision and enable the handling of large data volumes. While the current focus is on specific syndromes, as the review shows, we must prepare for a future in which new diseases may emerge due to climate change and other factors. This is not an easy challenge, given the high costs and workforce shortages that hinder the adoption of AI in the healthcare sector.^92^ However, this situation opens space for collaboration and innovation. We argue that Syndrome Discovery should evolve beyond a mere modernization of technological infrastructure to represent a true synergy between human expertise and artificial intelligence. In this context, AI/ML models function as advanced decision-support tools, assisting stakeholders in prioritizing surveillance indicators and rigorously evaluating the efficacy of public policies. Although the syndrome’s definitions may vary, it is possible to build flexible tools to adapt to different scenarios. The role of open science in syndromic surveillance is paramount. We need knowledge representation and AI solutions that can abstract local solutions and still perform well in different contexts, such as languages. By identifying the gaps in AI usage in this field, we encourage further exploration by researchers and aim to inspire the syndromic surveillance community to invest in open science. This involves publishing results and sharing code, models, and data whenever possible to foster transparency and collaboration in the research community.

## Author contributions

A.P.G.F. conceived the study, developed the methodology, conducted the literature search and analysis, and wrote the original article. A.A. contributed to the validation of selected articles, data interpretation, and article revision. A.U. provided epidemiological expertise, contributed to the interpretation of results, and reviewed the article. G.H. supervised the project, provided methodological guidance, and critically revised the article. All authors reviewed and approved the final version of the article.

## Declaration of interests

The authors declare no competing interests.

## Supplemental information

Document S1. Search strings

Document S2. PRISMA scoping review checklist
