# Supplementary material for: Advocating the potential of artificial intelligence for syndrome discovery in syndromic surveillance systems: A scoping review
Source: iScience. 2026 Feb 20;29(3):115103. doi: 10.1016/j.isci.2026.115103 (PMC13000536; doi:10.1016/j.isci.2026.115103)
Supplement: Document S1. Search strings [file mmc1.pdf]

iScience, Volume *29*

Supplemental information

Advocating the potential of artificial intelligence for syndrome discovery in syndromic surveillance systems: A scoping review

Ana Paula Gomes Ferreira, Aleksandar Anžel, Alexander Ullrich, and Georges Hattab

Supplementary Material

Search String Algorithm

General Definition: "syndromic surveillance" AND ((syndrom AND ("definition" OR "discovery" OR "classification")) OR "case definition") AND ("machine learning" OR "artificial intelligence" OR automat OR "deep learning" OR "natural language processing" OR "explainable AI" OR "reinforcement learning")

Google Scholar

URL: https://scholar.google.com/scholar?hl=en&as_sdt=0%2C5&q=%E2%80%9Csyndromic+surveillance%E2%80%9D+AND+%28%28syndrom*+AND+%28%E2%80%9Cdefinition%E2%80%9D+OR+%E2%80%9Cdiscovery%E2%80%9D+OR+%E2%80%9Cclassification%E2%80%9D%29%29+OR+%E2%80%9Ccase+definition%E2%80%9D%29+AND+%28%E2%80%9Cmachine+learning%E2%80%9D+OR+%E2%80%9Cartificial+intelligence%E2%80%9D+OR+automat+OR+%E2%80%9Cdeep+learning%E2%80%9D+OR+%E2%80%9Cnatural+language+processing%E2%80%9D+OR+%E2%80%9Cexplainable+AI%E2%80%9D+OR+%E2%80%9Creinforcement+learning%E2%80%9D+%29&btnG=

Search Query:

> "syndromic surveillance" syndrom definition|discovery|classification|"case definition" "machine learning"|"artificial intelligence"|automat|"deep learning"|"natural language processing"| "explainable AI"|"reinforcement learning"

PubMed

URL: https://pubmed.ncbi.nlm.nih.gov/?term=%22syndromic+surveillance%22+AND+%28+%28syndrom*+AND+%28%22definition%22+OR+%22discovery%22+OR+%22classification%22%29%29+OR+%22case+definition%22%29+AND+%28+%22machine+learning%22+OR+%22artificial+intelligence%22+OR+automat*+OR+%22deep+learning%22+OR+%22natural+language+processing%22+OR+%22explainable+AI%22+OR+%22reinforcement+learning%22%29

Search Query:

> "syndromic surveillance" AND ((syndrom* AND ("definition" OR "discovery" OR "classification")) OR "case definition") AND ("machine learning" OR "artificial intelligence" OR automat* OR "deep learning" OR "natural language processing" OR "explainable AI" OR "reinforcement learning")

Semantic Scholar

URL: https://www.semanticscholar.org/search?q=%22syndromic%20surveillance%22%20AND%20%28%28syndrom%20AND%20%28definition%20OR%20discovery%20OR%20classification%29%29%20OR%20%22case%20definition%22%29%20AND%20%28%22machine%20learning%22%20OR%20%22artificial%20intelligence%22%20OR%20automat%2A%20OR%20%22deep%20learning%22%20OR%20%22natural%20language%

20processing%22%20OR%20%22explainable%20AI%22%20OR%20%22reinforcement%20learning%22%29&sort=relevance

Search Query:

"syndromic surveillance" AND ((syndrom* AND (definition OR discovery OR classification)) OR "case definition") AND ("machine learning" OR "artificial intelligence" OR automat* OR "deep learning" OR "natural language processing" OR "explainable AI" OR "reinforcement learning")

OpenAlex

URL: https://openalex.org/works?page=1&filter=default.search:%22syndromic+surveillance%22+AND+((syndrom*+AND+(definition+OR+discovery+OR+classification))+OR+%22case+definition%22)+AND+(%22machine+learning%22+OR+%22artificial+intelligence%22+OR+automat*+OR+%22deep+learning%22+OR+%22natural+language+processing%22+OR+%22explainable+AI%22+OR+%22reinforcement+learning%22)

Search Query:

"syndromic surveillance" AND ((syndrom* AND (definition OR discovery OR classification)) OR "case definition") AND ("machine learning" OR "artificial intelligence" OR automat* OR "deep learning" OR "natural language processing" OR "explainable AI" OR "reinforcement learning")

Web of Science

URL: https://www.webofscience.com/

Search Query:

TS=("syndromic surveillance" AND ((syndrom* AND (definition OR discovery OR classification)) OR "case definition") AND ("machine learning" OR "artificial intelligence" OR automat* OR "deep learning" OR "natural language processing" OR "explainable AI" OR "reinforcement learning"))

Embase

URL: https://ovidsp.dc1.ovid.com/ovid-new-b/ovidweb.cgi

Search Query:

(syndromic surveillance and ((syndrom* and (definition or discovery or classification)) or "case definition") and ("machine learning" or "artificial intelligence" or automat* or "deep learning" or "natural language processing" or "explainable AI" or "reinforcement learning")).ti,ab.
